# Serotype distribution and antimicrobial resistance of *Streptococcus pneumoniae* in the Philippines, 2012–2018

**DOI:** 10.5365/wpsar-2021.12.4.834

**Published:** 2021-11-29

**Authors:** Sonia B. Sia, Marietta L. Lagrada, June M. Gayeta, Melissa Ana L. Masim, Jaywardeen P. Abad, Mariane A. Magbanua, Ferissa B. Ablola

**Affiliations:** a Research Institute for Tropical Medicine, Department of Health, Manila, Philippines.

## Abstract

**Objective:**

Data are scarce on the prevailing *Streptococcus pneumoniae* serotypes in the Philippines, including the relative antimicrobial resistance (AMR) of these bacteria. This study is designed to fill that gap by describing the serotype distribution and AMR of *S. pneumoniae* in the Philippines from 2012 to 2018.

**Methods:**

*S. pneumoniae* isolates from clinical specimens were collected through the Philippine Department of Health Antimicrobial Resistance Surveillance Program from 1 January 2012 to 31 December 2018. Identification and antimicrobial susceptibility testing (AST) were performed using conventional and automated methods (Vitek2 Compact Automated Machine). AST for penicillin, erythromycin, co-trimoxazole, ceftriaxone and levofloxacin was done following the Clinical and Laboratory Standard Institute recommendations. Serotyping was done through slide agglutination following the Denka Seiken slide agglutination method.

**Results:**

From a total of 307 isolates of *S. pneumoniae*, 32 serotypes were identified; the most frequently occurring were serotypes 1, 3, 5, 4, 18, 19A, 6B, 15 and 14. Many (*n* = 113, 36.53%) of the isolates were from those aged £5 years. Pneumococcal conjugate vaccine (PCV) coverage was as follows: PCV7 (32.69%), PCV10 (54.16%) and PCV13 (69.23%). The overall AMR of invasive *S. pneumoniae* isolates was low. Penicillin-resistant serotypes were 14, 19, 24, 4, 5, 1, 15, 6 and 32.

**Discussion:**

With the inclusion of PCV13 in the National Immunization Program, continued monitoring of the prevailing serotypes of *S. pneumoniae* isolates in the Philippines is needed to guide disease and AMR control measures.

*Streptococcus pneumoniae* poses a serious public health concern because it causes a wide range of diseases including otitis media, septicaemia, meningitis and pneumonia. The World Health Organization (WHO) reports that pneumonia accounted for 15% of mortalities among children aged £5 years globally in 2017. (*1*) *S. pneumoniae* was identified as one of the leading causes of pneumonia in the 2016 Global Burden of Disease report. (*2*) Invasive pneumococcal disease (IPD), defined as infection of normally sterile sites of the body with *S. pneumoniae*, most frequently affects children aged < 2 years, adults aged (*3*)65 years and immunocompromised patients. (*3*, *4*) In the Philippines, a study in Regions VI, VII and VIII determined that there were 89 221 children aged < 5 years with pneumonia who were seen and 85 923 who were given medication from January to December 2012. (*5*)

At present, more than 94 different pneumococcal serotypes have been classified based on the unique polysaccharide characteristics and composition expressed in the capsule. (*6*) Serotype 19A was the most commonly identified serotype in the regions of East Africa, Asia Pacific, United States of America (USA), Europe and North America in 2007–2015. (*7*) Serotypes 6B, 14 and 19F were the predominant causes of IPD among children in the Africa–Eastern Mediterranean region, whereas serotypes 1 and 14 were prevalent in Europe and Latin America.

The threat of emerging antimicrobial resistance (AMR) among *S. pneumoniae* serotypes worldwide was recognized as early as the 1980s. Antimicrobial susceptibility profiling of *S. pneumoniae* has played a significant role in the treatment of patients and in mapping AMR for large-scale epidemiology studies. Specific *S. pneumoniae* serotypes have been associated with resistance to specific antimicrobial agents; for example, serotypes 19F, 14, 23F, 9V and 6B have been found to be resistant to penicillin and macrolides. (*3*)

Data are lacking on the prevailing pneumococcal serotypes in the Philippines, including their resistance to specific antimicrobials. This study therefore describes the distribution and AMR of *S. pneumoniae* serotypes in the Philippines from 2012 to 2018.

## Methods

### Bacterial isolates

*S. pneumoniae* isolates from invasive clinical specimens were collected through the Philippine Department of Health Antimicrobial Resistance Surveillance Program (DOH-ARSP) from 1 January 2012 to 31 December 2018. The DOH-ARSP is a laboratory-based AMR surveillance programme with 24 sentinel sites representing 16 of the 17 geopolitical regions in the country. There are two private hospitals among the eight sentinel sites in the National Capital Region, but all other sentinel sites are regional government hospitals that cater to their respective geopolitical regions. All are tertiary hospitals with bed capacity ranging from 50 to 1500, with many being in the 300–500 range.

Case finding for DOH-ARSP is based on priority specimens sent routinely to sentinel site laboratories for clinical purposes. Thus, sampling in the present study is largely based on diagnostic practices of the sentinel site clinicians. All *S. pneumoniae* isolates grown from invasive clinical specimens were included in the present study. Cumulative overall analyses were done for all isolates, with a focus on the most vulnerable age groups, that is, those aged £5 years and those aged (*3*)65 years.

### Bacterial identification and antimicrobial susceptibility testing

*S. pneumoniae* isolates were cultured by the sentinel sites from invasive clinical samples based on the WHO Manual for the laboratory identification and antimicrobial susceptibility testing of bacterial pathogens of public health importance in the developing world. (*8*) Isolates were then sent to the implementing laboratory of the DOH-ARSP for confirmation of identification and antimicrobial susceptibility testing (AST) and for serotyping. Confirmation of identification and AST of isolates were performed using the Vitek2 Compact Automated Machine (bioMérieux). AST for penicillin, erythromycin, co-trimoxazole, ceftriaxone and levofloxacin was done following the method described by the Clinical and Laboratory Standard Institute (CLSI). (*9*) Results were managed and analysed using WHONET 5.6, a Windows-based database software that facilitates analysis of AST. In computing percentage resistance, only the first isolate per patient per calendar year was included.

### Serotyping

*S. pneumoniae* isolates were serotyped through slide agglutination following the Denka Seiken slide agglutination method as described by Denka Seiken Co., Ltd (*10*) Because of local unavailability of factor sera, typing within serogroups that contained multiple serotypes was not done.

## Results

### IPD serotype distribution

A total of 307 isolates of *S. pneumoniae* were collected from patients with IPD in the 7-year study period. The age range was 0–93 years. Most of the isolates were from blood (*n* = 286, 93.15%) and cerebrospinal fluid (*n* = 21, 6.84%). About a third (*n* = 113, 36.80%) of the isolates were from the £5 years age group followed by the age groups 18–64 years (*n* = 111, 36.15%), (*3*)65 years (*n* = 55, 17.91%) and 6–17 years (*n* = 28, 9.12%).

Thirty-two serotypes were identified, with the most frequently occurring being serotypes 1, 6, 3, 5, 4, 18, 23, 12, 15 and 2 (**Table 1**). These 10 serotypes made up 71% of the total isolates. Due to local unavailability of typing sera, no typing was done for serogroups 6, 18, 19 and 23.

**Table 1:** Frequency of *Streptococcus pneumoniae* serotypes in the Philippines, 2012–2018 (*n* = 307)

Serotype	2012 *n* = 7	2013 *n* = 20	2014 *n* = 33	2015 *n* = 51	2016 *n* = 63	2017 *n* = 59	2018 *n* = 74	TOTAL	%
4	1	1	4	5	3	3	6	23	7.49
6	-	1	5	8	7	3	3	27	8.79
9	-	-	1	-	4	-	3	8	2.61
14	2	2	-	1	-	5	3	13	4.23
18	-	1	2	5	1	8	4	21	6.84
19	-	-	-	1	4	0	4	9	2.93
23	1	2	2	2	4	6	4	21	6.84
1	-	5	5	7	9	6	6	38	12.37
5	2	2	8	2	3	1	5	23	7.49
7	-	-	-	1	3	1	1	6	1.95
3	-	1	1	3	4	9	8	26	8.46
2	1	2	1	2	4	1	1	12	3.90
10	-	-	-	1		1	3	5	1.63
11	-	-	-	2	1	-	-	3	0.98
12	-	-	-	2	1	1	1	5	1.63
15	-	-	1	2	6	2	2	13	4.23
20	-	-	1	1	1	1	3	7	2.28
22	-	-	-	-	3	1	1	5	1.63
33	-	1	-	1	2	-	-	4	1.3
16	-	-	-	-	-	2	5	7	2.28
21	-	-	-	-	-	1	-	1	0.33
24	-	-	-	-	2	-	2	4	1.3
25	-	-	-	1	-	1	1	3	0.98
28	-	-	-	1	-	1	-	2	0.65
29	-	-	-	2	-	2	2	6	1.95
31	-	-	-	1	1	-	-	2	0.65
32	-	1	-	-	-	-	-	1	0.33
34	-	1	1	-	-	-	3	5	1.63
35	-	-	-	-	-	-	2	2	0.65
39	-	-	-	-	-	1	-	1	0.33
40	-	-	1	-	-	2	-	2	0.65
46	-	-	-	-	-	2	-	1	0.33
**TOTAL**								**307**	

Legend:

-

PCV7

Serotypes: 4, 6B, 9V, 14, 18C, 19F, 23F

-

-

PCV10

Serotypes: PCV7 + 1, 5, 7F

-

-

PCV13

Serotypes: PCV10 + 3, 6A, 19A

-

-

PPSV23

Serotypes: 1, 2, 3, 4, 5, 6B, 7F, 8, 9N, 9V, 10A, 11A, 12F, 14, 15B, 17F, 18C, 19A, 19F, 20, 22F, 23F, 33F

The overall PCV coverages of the serogroups identified in this study were as follows: PCV7 (39.73%), PCV10 (59.60%) and PCV13 (68.07%). There were 37 isolates (12%) with serotypes not included in PCVs and PPVs (non-vaccine types) (**Table 1**).

Among patients aged £5 years, the most common serotypes were 6 (*n* = 16, 14.15%), 18 (*n* = 12, 10.61%) and 14 (*n* = 10, 8.85%), all covered by PCVs. The overall PCV coverages of serotypes from this age group were 54.86% for PCV7, 66.37% for PCV10 and 70.79% for PCV13. A total of 11% of the isolates among this age group were non-vaccine serotypes.

Among isolates from older adults, those aged (*3*)65 years, the most frequent were serotypes 3 (*n* = 10, 18.18%), 4 (*n* = 5, 9.09%) and 1 (*n* = 5, 9.09%), which are all covered by PCV. Serotype 3 (present in PCV13 but not in PCV7 and PCV10) was consistently seen in this age group from 2014 to 2018. The overall vaccine coverages in this age group were PCV7 (29.09%), PCV10 (50.90%), PCV13 (69.09%) and PPV23 (89.09%). Only 7% (4/55) were non-vaccine serotypes.

There were only 28 isolates from patients aged 6–17 years, with the most common being serotypes 1 (*n* = 5, 17.85%) and 18 (*n* = 4, 14.28%), both covered by the conjugate vaccines. Among the isolates from patients aged 18–64 years, the most common were serotypes 1 (*n* = 21, 18.91%), 4 (*n* = 12, 10.81%) and 23 (11, 9.90%), all of which were covered by PCVs. Only 15% (11/111) were non-vaccine serotypes.

**Figure 1:**
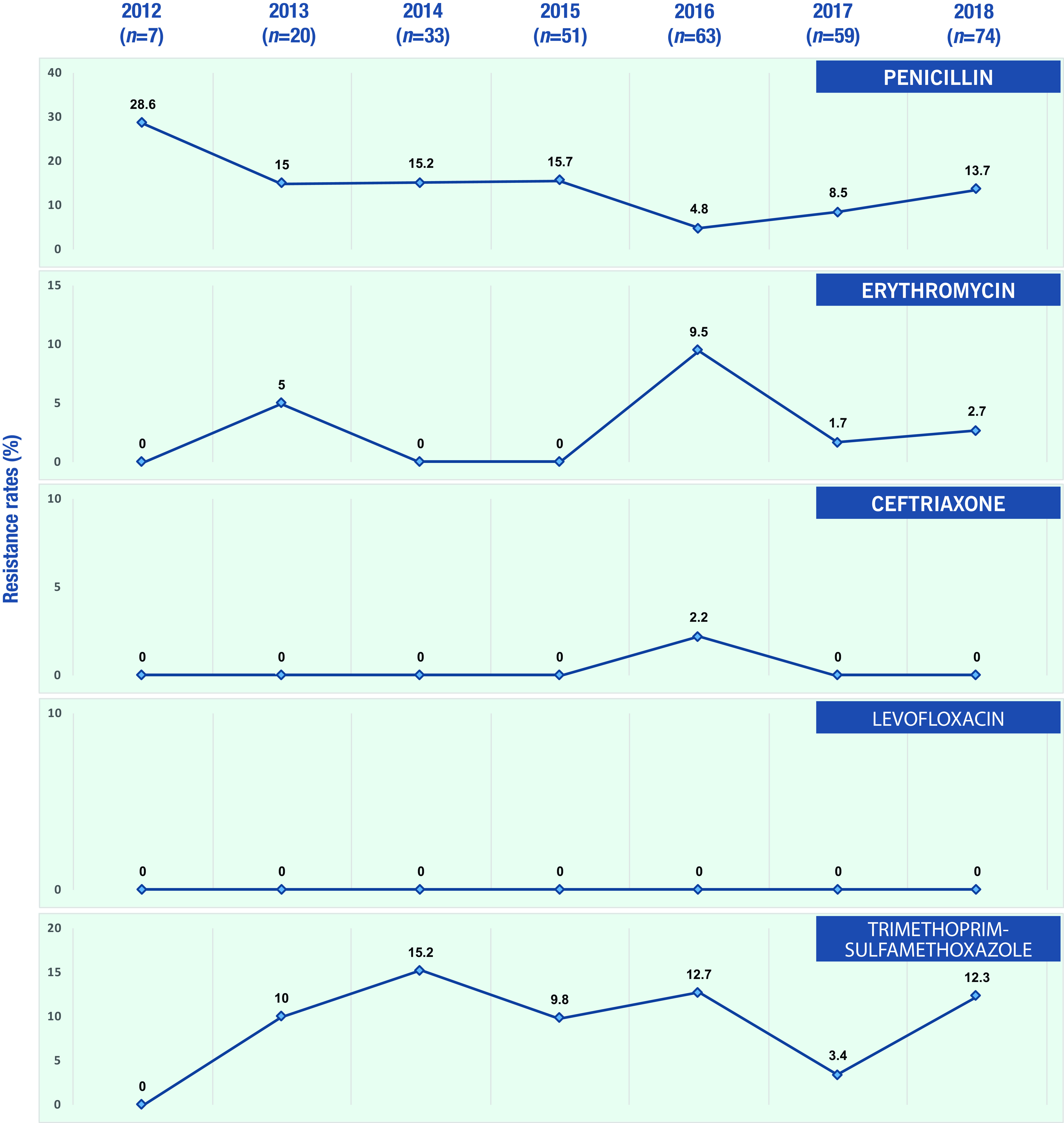
EYearly antimicrobial resistance rate of penicillin, erythromycin, ceftriaxone, levofloxacin and trimethoprimsulfamethoxazole in the Philippines, 2012–2018

The overall cumulative resistance rate to antibiotics of interest among the invasive *S. pneumoniae* isolates in this study was low. Resistance to penicillin (meningitis breakpoint) was highest at 14.57%, followed by co-trimoxazole (9.06%), erythromycin (2.7%) and ceftriaxone (0.31%). No resistance to levofloxacin was seen in this study. The only distinct trend in yearly AMR rates was seen for penicillin, with a 2-year successive increase in 2017 and 2018. However, given the relatively low number of isolates, these increases were not statistically significant (**Fig. 1**).

[**insert** Figure 1]

### Antimicrobial resistance

There were 34 penicillin-resistant isolates in the study, of which 56% (19/34) were from patients aged £5 years and were of serotypes 14 (*n* = 8, 42%), 19 (*n* = 4, 21%), 6 (*n* = 2, 10%), 1, 4, 15, 33 and 24 (*n* = 1 each, 5% each). PCV coverage of penicillin-resistant isolates from this age group was 84%, with three non-PCV serotypes: serotypes 15, 33 and 24. Serotypes of penicillin-resistant isolates from other age groups are shown in **Table 2**.

**Table 2:** Distribution of penicillin-resistant isolates by age and serotype

Age group	Number of isolates	Serotypes
£5	19 (55.9%)	4, 6, 14, 19, 1, 15, 33, 24
6–17	2 (5.9%)	14, 5
18–64	10 (29.4%)	4, 19, 1, 5, 15, 24
^3^65	3 (8.8%)	19, 5, 32
Total	34	-

Of the 21 isolates resistant to co-trimoxazole, most (*n* = 13, 62%) were from patients aged £5 years and were of serotypes 6, 14, 19, 23 and 5. PCV coverage of such isolates in this age group was 100%.

There were few erythromycin-resistant isolates in this study, with most (4/7, 57%) coming from the adult population (18–64 years) and only two isolates from children aged £5 years. The erythromycin-resistant isolates were of serotypes 6, 1, 23 and 24, with the non-vaccine serotype 24 being the most common type (3/7, 43%).

One serotype 24 isolate from a male child aged 6 months was reported in 2015 to be resistant to ceftriaxone. This non-vaccine type isolate was noted to also be resistant to erythromycin and penicillin. There was no report of any similarly resistant phenotype in the succeeding years.

Among the 10 serotype 14 isolates from patients aged £5 years, eight (80%) were penicillin-resistant. Further, of the nine serotype 19 isolates, six (67%) were penicillin-resistant, with four of these isolated from patients aged £5 years.

## Discussion

### IPD serotype distribution

Pneumococcal serotypes vary in prevalence, age group infected, geographical distribution and AMR pattern. Local IPD serotypes identified in this study (serotypes 1, 3, 4, 6, 14, 18 and 23) resemble the dominant IPD serotypes worldwide, including 1, 3, 4, 14, 6A, 6B, 7F, 8, 18C, 19F, 9V and 23F. (*11*) Serotype 1 was present yearly in all age groups and accounted for the greatest number of isolates across each age group. Serotypes 4 and 5 were also observed in all age groups and were present in each year of the study period. Of the 32 serotypes identified in this study, seven have not been reported previously in local studies: serotypes 10 and 11, which are covered by PPV, and five non-vaccine types, 21, 32, 35, 40 and 46. The IPD serotype distribution in this study relies on the diagnostic practices of sentinel site clinicians. This study does not provide data on the proportion of IPD cases that had isolates for testing; however, it does provide information on the serotype distribution and AMR of *S. pneumoniae* in the Philippines.

Following the introduction of PCV13 in Asian countries in 2009, the pattern of vaccine serotype coverage and predominant IPD serotypes detected has changed. PCV7 serotype coverage reduction was noted to be 30–34% in the Republic of Korea, Hong Kong Special Administrative Region SAR (China) and China, Taiwan (China). (*12*) In the PCV13 period, the most prevailing serotype was 19A in Japan, 3 in China, Taiwan (China) and 15 in China. This is in contrast with the results of the present study where serotypes 1 (*n* = 38, 12.37%) and 6 (*n* = 27, 8.79%) predominated. The difference in the prevailing serotypes across the region could be influenced by the presence of antibiotic-resistant strains, immunogenicity of each conjugate in different populations and a mismatch between serotype variants present in a country and the available strains used in vaccine preparation. (*12*)

The overall PCV13 coverage of 68.07% in the present study is lower than was found in a previous local 8-year study (2004–2011), where it was 73.8%. (*13*) This may be due to the larger number of isolates in the present study. The overall PCV13 coverage among isolates from patients aged £5 years in the present study was 70.79% – lower than the reported 80.4% in Hong Kong Special Administrative Region SAR (China) and 93.1% in China, Taiwan (China). (*12*)

PCV13 was included in the country’s National Immunization Program for children aged £5 years in 2015, with low vaccination coverage ranging from 30% to 60% in 2015–2019. (*1*) However, there was no noted decrease in PCV13 coverage among the isolates from this age group in 2015, with PCV13 coverage ranging from 68% in 2016 to 79% in 2018. Continuous surveillance of *S. pneumoniae* serotypes can track changes to prevailing serotypes, especially if vaccination coverage improves.

Among the isolates from those aged (*3*)65 years, serotypes included in PCV13 (3, 1, 4, 18, 6) were the most common. These findings support the 2018 local immunization recommendation of administering PCV13 to this age group. (*14*)

### Antimicrobial resistance

The cumulative resistance rates of pneumococci in the present study were low, ranging from 0% for levofloxacin to 14.57% for penicillin. This is lower than reported elsewhere in Asia, including values for penicillin resistance among pneumococci causing IPD in 17 Chinese cities of 51.6% (455/881) and erythromycin resistance of 95.2% (839/881) during 2011–2016. (*15*) A study from the Asian Network for Surveillance of Resistant Pathogens reported pneumococci resistance rates of 1.7%, 0.4%, 1.5% and 13.4% for levofloxacin, moxifloxacin, gatifloxacin and ciprofloxacin, respectively. (*16*) The resistance rates to ceftriaxone among 10 hospitals in China were 8.2% and 18.1% among non-meningeal and meningeal isolates, respectively. (*17*) A medical research institute in Malaysia reported that 35.9% of the total pneumococcal isolates (663/1847) from a paediatric population was resistant to co-trimoxazole. (*18*)

The most common multidrug resistance pattern observed in this study was a penicillin-erythromycin-co-trimoxazole combination (*n* = 3). This combination was also found in 6/125 resistance patterns in a multicentre retrospective study in China. (*19*)

### Serotypes and AMR

Specific pneumococcal serotypes are known to be associated with certain antibacterial resistance. (*19*) Worldwide, penicillin resistance was observed among serotypes 6A, 6B, 9V, 14, 19A, 19F, 23A and 35B, with the most resistant serotypes being 19A (28.1%), 19F (19.0%) and 35 (16.7%). (*20*, *21*) Three of these serotypes were identified among the penicillin-resistant isolates in the present study – 6, 14 and 19A – all of which are covered by PCV13. With four of the six isolates from patients aged £5 years, vaccination with PCV13 could prevent penicillin resistance among pneumococci in this age group. Interestingly, all four of the serotype 24 isolates – a non-vaccine type – from the £5 year and the 16–84 year age groups in this study were penicillin-resistant. Monitoring this serotype is recommended to guide control measures against the spread of penicillin-resistant pneumococci.

Erythromycin resistance has been observed among serotypes 6A, 6B, 9V, 14, 15A, 19A and 19F, (*22*) and for serotypes 6 (21.8%) and 14 (41.9%) among children aged £5 years. (*19*) Results from this study differ, with serotypes 1 (2.6%), 6 (7.4%), 23 (14%) and 24 (100%) being erythromycin-resistant isolates. Co-trimoxazole-resistant isolates in the present study were from serotypes 6 (45%) and 19 (19.35%), similar to results reported for co-trimoxazole-resistant serotypes (serotype 6B) from Malaysia. (*21*)

Although particular serogroups have been associated with resistance to specific antibiotics recently, it is possible that serotype profiles of resistant pneumococci will change through the years because the genes encoding the capsular serotype can be exchanged and acquired. (*23*) This, as well as the potential for serotype replacement and switching serotypes within the conjugate vaccines, suggests that monitoring pneumococci serotypes is required in the Philippines. Whole genome sequencing could also be considered for monitoring pneumococci serotypes, given that AMR among pneumococci is usually clonal in origin.

## Conclusion

The *S. pneumoniae* serotypes in the present study are largely similar to those prevailing worldwide. The most common serotypes and serogroups observed in this study were serotypes 1, 6, 3, 5, 4, 18, 23, 15, 14 and 2. PCV coverage among patients aged £5 years across the 7-year study has not decreased, even after the inclusion of PCV13 in the National Immunization Program. The AMR rates of *S. pneumoniae* to penicillin, erythromycin, ceftriaxone, co-trimoxazole and levofloxacin remained low. The specific antibiotic-resistant serotypes observed in this study were similar to those in other Asian countries. All serotype 24 isolates, a non-vaccine type, were found to be resistant to penicillin and erythromycin. With the inclusion of PCV13 in the National Immunization Program, continued monitoring of the prevailing serotypes of *S. pneumoniae* isolates in the Philippines is needed to guide disease and AMR control measures.
